# Bearing fault detection by using graph autoencoder and ensemble learning

**DOI:** 10.1038/s41598-024-55620-6

**Published:** 2024-03-03

**Authors:** Meng Wang, Jiong Yu, Hongyong Leng, Xusheng Du, Yiran Liu

**Affiliations:** 1 School of Information Science and Engineering, Xinjiang University, Urumqi, 830046 China; 2 School of Software, Xinjiang University, Urumqi, 830046 China

**Keywords:** Bearing fault detection, Graph neural network, Ensemble learning, Outlier detection, Intelligent fault detection, Machine learning, Electrical and electronic engineering, Mechanical engineering

## Abstract

The research and application of bearing fault diagnosis techniques are crucial for enhancing equipment reliability, extending bearing lifespan, and reducing maintenance expenses. Nevertheless, most existing methods encounter challenges in discriminating between signals from machines operating under normal and faulty conditions, leading to unstable detection results. To tackle this issue, the present study proposes a novel approach for bearing fault detection based on graph neural networks and ensemble learning. Our key contribution is a novel stochasticity-based compositional method that transforms Euclidean-structured data into a graph format for processing by graph neural networks, with feature fusion and a newly proposed ensemble learning strategy for outlier detection specifically designed for bearing fault diagnosis. This approach marks a significant advancement in accurately identifying bearing faults, highlighting our study's pivotal role in enhancing diagnostic methodologies.

## Introduction

Rotating machinery plays a crucial role in automation and industrial processes. However, motor failures can result in high maintenance costs, accidents, and even fatalities due to mishandling, adverse conditions, and wear and tear^1,2^. Among various issues that can lead to failures, bearing failures are the most common, accounting for 30–40% of total failures^3,4^. The degree of damage in rolling bearings significantly affects the effectiveness of fault detection methods. In the early stages, minor wear or defects may cause subtle signal anomalies that are often difficult to detect due to background noise. As the damage worsens, these signal anomalies become more pronounced, making it easier to identify the faults but also indicating potentially advanced issues. The severity of the damage directly affects the characteristics of the vibration signal, with minor wear mainly affecting high-frequency components and more significant damage impacting both high and low frequencies. This calls for different detection approaches to accurately diagnose faults. Furthermore, severe damage introduces increased signal complexity, where symptoms of multiple fault types can be observed in a single bearing. To address this complexity, advanced analytical methods, such as sophisticated machine learning models, are needed to decipher complex signal patterns. This highlights the critical role of the extent of damage in the precision and reliability of bearing fault detection strategies. Existing diagnostic methods face challenges in timely and accurate bearing fault detection, which can lead to motor failures and severe injuries. These methods often focus on the characteristics of individual objects and neglect the relationships among them, making it difficult to identify abnormal samples mixed with normal ones and accurately reflect the fault state of the bearings. Existing diagnostic methods face challenges in timely and accurate bearing fault detection, which can lead to motor failures and severe injuries. These methods often focus on the characteristics of individual objects and neglect the relationships among them, making it difficult to identify abnormal samples mixed with normal ones and accurately reflect the fault state of the bearings. To address this, we propose an innovative bearing fault diagnosis method called BFDGE (Bearing Fault Detection using Graph Neural Networks and Ensemble Learning), leveraging advancements in machine learning, specifically graph neural networks and ensemble learning. Our method has three main contributions: (i) a stochasticity-based graph construction method to convert vibration signals into graph-structured data, allowing for better information gathering from neighboring objects, (ii) the integration of graph neural networks with ensemble learning, introducing a novel ensemble learning strategy to enhance model robustness and stabilize detection outcomes, and (iii) empirical validation using public datasets from Case Western Reserve University (CWRU) and Xi'an Jiaotong University (XJTU), demonstrating the effectiveness of our method in distinguishing anomalous signals within normal signals and enabling more efficient and accurate fault identification.

## Related work

Deep learning has been widely applied in various domains^5,6^, including bearing fault diagnosis. Vibration analysis is a commonly used technique for diagnosing bearing faults^7–11^. By monitoring the vibration signal of a bearing and analyzing its spectrum and characteristics, valuable information such as the type, extent, and location of the fault can be determined. Previous research has shown that vibration analysis is effective in detecting bearing faults, especially in the early stages with high accuracy. Xu et al.^12^ proposed a method using autocorrelation envelopes to detect early rolling bearing faults. The underlying concept of this method is that the autocorrelation of a bearing's vibration signal changes when a fault occurs, enabling early detection. However, this approach requires more complex signal processing and feature extraction, as well as comprehensive training and optimization of the classifier for optimal performance. Liu et al.^13^ proposed an empirical wavelet thresholding method based on vibration analysis for detecting large wind turbine blade bearing faults. The objective of this method is to identify early faults in blade bearings by analyzing blade vibration signals. However, this method has the drawback of requiring complex wavelet decomposition and thresholding of the signal, as well as the need for thorough parameter optimization to achieve optimal performance. Wang et al.^14^ improved the accuracy and reliability of bearing fault detection by combining time and frequency domain information. However, this method has some drawbacks, including the need for complex signal processing and feature extraction, as well as thorough training and optimization of the classifier to achieve optimal performance. Li et al.^15^ presented an enhanced method for detecting rolling bearing faults, which combines sparse coding systolic denoising and fast spectral correlation. This approach utilizes fast spectral correlation to extract frequency domain features from the processed signal, thereby enhancing fault detection performance. Tao et al.^16^ proposed a method for detecting bearing faults that utilizes wavelet transform and generalized Gaussian density modeling. The objective of this approach is to identify early faults in bearings by analyzing their vibration signals. With the rise of artificial intelligence, machine learning has become a widely used technique in machine fault diagnosis^17–24^, giving birth to a new field called intelligent fault diagnosis (IFD). In recent years, there has been a growing number of researchers dedicated to IFD. Zhang et al.^25^ proposed a method for diagnosing bearing faults based on deep convolutional neural networks (DCNN), which can achieve more accurate fault diagnosis results in noisy environments and under various workloads by using a novel training approach and optimizing the network structure. However, the data augmentation techniques employed in this method may introduce some noise and unnecessary complexity, despite their ability to enhance the quantity and diversity of the training data. Xia et al.^26^ introduced a novel approach for diagnosing faults in rotating machinery by combining multiple sensors and convolutional neural network (CNN) techniques. This method utilizes different types of signals collected from multiple sensors and employs CNN to accurately diagnose faults in rotating machines. However, the fault classification in this method may be limited to predefined fault types, preventing the diagnosis of unknown faults. Qian et al.^27^ proposed a novel migration learning method for fault diagnosis in rotating machinery, especially under diverse operating conditions. This method utilizes a pre-trained convolutional neural network (CNN) model for migration learning. However, the CNN model may abstract too much from the data's details and local features, resulting in the inability to capture significant features in certain cases. Fu et al.^28^ presented a method for diagnosing rolling bearing faults and selecting features. They used empirical mode decomposition (EMD) and an optimized Elman AdaBoost algorithm. This method decomposes the vibration signal into intrinsic modal functions (IMFs) using EMD, and then extracts and selects energy features from these IMFs. The selected features are classified and diagnosed using an optimized Elman neural network and the AdaBoost algorithm, allowing for automated diagnosis of rolling bearing faults. However, it is important to note that the use of an Elman neural network and the AdaBoost algorithm in this method may have limitations in terms of overfitting and generalization. In a different study, Spyridon et al.^29^ introduced a method for detecting and identifying rolling bearing faults using an attention mechanism and a dense convolutional neural network. Their proposed method aims to achieve efficient and precise detection and identification of bearing faults by analyzing rolling bearing vibration signals through convolutional neural networks. Another approach was proposed by Tobias et al.^30^, who developed a method for detecting bearing faults based on deep neural networks and weighted integrated learning. Their method specifically focuses on analyzing multi-motor phase current signals to achieve efficient and precise bearing fault detection. Liu^31^ conducted a study where vibration signals from rolling bearings were collected and cyclic spectrum analysis techniques were applied to extract the features. The method then describes and represents these features using support vector data description. Furthermore, a semi-supervised learning model was developed to classify and diagnose the features for early detection of rolling bearing faults. In a similar vein, Khorram et al.^32,33^ utilized the vibration signal of a gear bearing as input and employed a convolutional neural network for feature extraction and filtering. The resulting feature sequence was then inputted into a long and short-term memory network for time series analysis and fault diagnosis. This approach enables direct learning of features and patterns from raw data, eliminating the need for manual feature extraction and selection. These studies highlight the wide range of promising applications of machine learning techniques in the field of intelligent fault diagnosis. Ensemble learning has gained popularity in the field of machine learning^34–39^. One well-known ensemble learning strategy is Adaboost^40^, which involves iteratively training multiple weak classifiers with weighted samples and combining them into a strong classifier to improve classification accuracy. Zhou et al.^41^ proposed an ensemble domain-based adaptive learning algorithm that enhances the performance and generalization of the model by adaptively adjusting the overall model weights and structure. However, this algorithm has a high time complexity and may require more computational resources and time. In contrast, Alam et al.^42^ proposed a neural network algorithm based on dynamic ensemble learning. This algorithm adaptively adjusts the model structure to suit different data types and features. However, it may have limitations in dealing with large and complex data. Webb et al.^43^ introduced a multi-strategy ensemble learning approach that combines different ensemble learning techniques to achieve better performance and generalization capability. This method is able to adapt to different data types and task requirements, and is robust and scalable. Additionally, Xu et al.^39^ proposed a forest fire detection system that utilizes various machine learning algorithms, such as random forests, support vector machines, and neural networks, to construct detection models and provide high accuracy. However, implementing this system may require certain technical expertise in machine learning and software development.

Outlier detection, also known as anomaly detection, is a technique in machine learning and data mining that aims to identify data objects exhibiting different behavior than the predicted data. These objects, referred to as outliers, fundamentally differ from the normal behavior pattern of the data. Unlike noise, which represents random errors and variances in observed variation, outliers in production machines deviate significantly from the rest of the data. Bearing failure itself is considered an anomaly, making outlier detection a relevant research direction in machine learning. Therefore, we can utilize outlier detection techniques to address the problem of bearing failure. Additionally, ensemble learning, which combines multiple algorithms, can enhance the performance, robustness, and stability of models. In this study, we applied well-established ensemble learning techniques from the machine learning field to the domain of bearing fault diagnosis. Specifically, we selected five mature outlier point detection algorithms as the base detectors.

Graph AutoEncoder^44^ (GAE) is a widely used graph neural network-based method for outlier detection. It sorts data objects in descending order and calculates outlier factors to determine outliers. AutoEncoder^45^ (AE) is a type of multilayer feedforward neural network where the number of nodes in the input and output layers are equal, and the hidden layer has a relatively small number of nodes. AutoEncoder is employed for outlier detection by learning the feature representation of normal data and identifying abnormal data that significantly deviates from normal data. Local Outliers Factor^46^ (LOF) is an unsupervised anomaly detection algorithm that measures the local density deviation of a given data point in relation to its neighborhood. The degree of anomaly of each point is determined by comparing its density with that of its neighbors. Connectivity-Based Outlier Factor^47^ (COF) is another algorithm used for outlier detection, which assesses the degree of outliers based on the connectivity between data points. The COF value of each data point is calculated by measuring the connectivity between the data point and its nearest neighbor, as well as the average connectivity among all points in its neighborhood. K-Nearest Neighbors^48^ (KNN) is a classical outlier detection algorithm that assigns an outlier score to each data point based on its K-nearest neighbor data points. The core idea behind KNN is that outliers have denser neighborhoods, while normal data points have sparser neighborhoods. In KNN, a data point's K nearest neighbor data points should have relatively small distances, whereas the distance between that data point and its K + 1 nearest neighbor data points should be relatively large.

## Methodology

**Figure 1:**
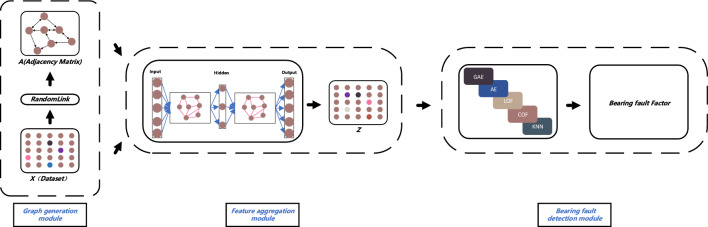
The entire structure for bearing fault detection.

In this study, we employed ensemble learning and graph neural network techniques, which are commonly used in machine learning, to address the issue of bearing fault diagnosis. Our model consists of three modules: a graph generation module, feature fusion module, and bearing fault detection module. This section provides a detailed description of these three modules. The core idea of our method is to convert the original Euclidean dataset into an adjacency matrix ***A*** using the randomness-based combination module. Then, the original dataset ***X*** and the adjacency matrix ***A*** are inputted into the feature aggregation module to generate aggregated adjacency features, which are used to obtain the matrix ***Z***. The matrix ***Z*** is then fed into an ensemble learning-based anomaly detection module (COF, LOF, GAE, AE, and KNN) for bearing fault detection, resulting in the anomaly matrix. The top-S base detectors with better detection ability are selected in descending order, and the final outliers of each node are obtained by averaging the outliers obtained from the top-S base detectors. The structure of BFDGE is shown in Fig. 1.

### Random connections-based graph construction method

Graph generation is the process of converting each object in a Euclidean dataset into a node and organizing them into graph data. This process involves the following steps: a) importing the dataset ***X*** and initializing its adjacency matrix ***A***. b) assigning a value of 1 to all diagonal elements of matrix ***A*** to represent self-connections of each node. c) selecting one object as the root node, randomly choosing ***k*** objects from the remaining set, and connecting the root node to these nodes by creating directed edges.

#### Euclidean distance calculation

The Euclidean distance, also known as the Euclidean metric, represents the distance between two points in Euclidean space. In the higher dimensional Euclidean space, the Euclidean distance is calculated by summing the squared differences of each individual dimension n.

1 $$ d(\mathop x\nolimits_{i} ,\mathop x\nolimits_{j} ) = \sqrt {(\mathop x\nolimits_{i1} - \mathop x\nolimits_{j1} )^{2} + \cdots + \mathop {(\mathop x\nolimits_{in} - \mathop x\nolimits_{jn} )}\nolimits^{2} } $$

Assuming *X*_*i*_, *X*_*j*_ ∈ *X* and *X*_*i*_ ≠ *X*_*j*_, a root node *i* is selected and a parameter *K* is set. *K* nodes are randomly selected from *X*. If the selected K nodes include the root node *i*, it is re-selected. Then, the selected *K* nodes are stored in a random set of neighbors *Nk(Xi)*.

#### Constructing the adjacency matrix

The Euclidean distance from root node *i* to any point in its set of random neighbors *N*_*k*_(*X*_*i*_) is normalized to the weight of the directed edge from root node *i* to that point.

2 $$ W(X_{i} ,X_{j} ) = \left\{ {\begin{array}{*{20}l} {\frac{{d(X_{i} ,X_{j} )}}{{\sum\nolimits_{j = 1}^{k} {d(X_{i} ,X_{j} )} }},} \hfill & {\quad X_{j} \in N_{k} (X_{i} )} \hfill \\ {0,} \hfill & {\quad X_{j} \notin N_{k} (X_{i} )} \hfill \\ \end{array} } \right. $$

When *X*_*j*_ ∉ *N*_*k*_(*X*_*i*_),the weight between* X*_*i*_ and *X*_*j*_ is 0.when *X*_*j*_ ∈ *N*_*k*_(*X*_*i*_), the weights between* X*_*i*_ and *X*_*j*_ are as shown in (2). We represent the resulting graph through the adjacency matrix *A* as follows:

3 $$ A = \left\vert\begin{array}{*{20}c}  1 & {W(X_{2} ,X_{1} )} & \cdots & \cdots & {W(X_{m} ,X_{1} )}  \\  {W(X_{1} ,X_{2} )} & 1 & \cdots & \cdots & {W(X_{m} ,X_{2} )}  \\ \cdots & \cdots & {W(X_{i} ,X_{j} )} & \cdots & \cdots  \\  \cdots & 0 & \cdots & \cdots & {W(X_{m} ,X_{i} )}  \\  {W(X_{1} ,X_{m} )} & {W(X_{2} ,X_{m} )} & \cdots & \cdots & 1  \\ \end{array}\right\vert $$

**Figure 2:**
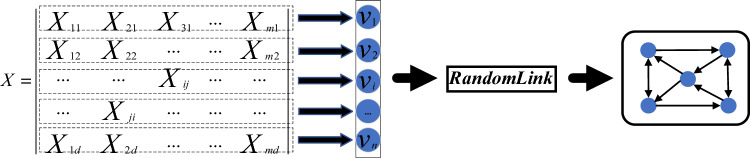
Graph generation.

In order to preserve the characteristics of the root node, the diagonal of the adjacency matrix is set to 1. Please note that *W(X*_*i*_*,X*_*j*_*)* does not necessarily equal *W(X*_*j*_*,X*_*i*_*)*. The structure of graph generation model is shown in Fig. 2.

**Algorithm 1:**
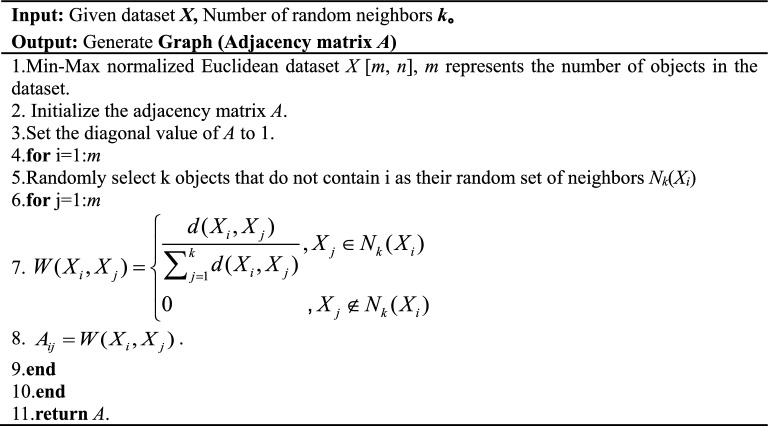
Graph Generation.

### Aggregating neighbor node characteristics via GNN

In this study, we propose a graph autoencoder (GAE)-based approach for fusing node features in a Euclidean dataset. Our approach generates a new matrix ***Z*** by aggregating neighboring node features. The objective is to address the limitations of existing outlier detection algorithms, specifically in identifying outliers within normal target regions or those mixed around dense clusters. By reconstructing the original dataset, we can accurately isolate these outliers, thereby improving the accuracy and robustness of outlier detection and facilitating downstream tasks. The primary advantage of our approach lies in its adaptive ability to capture the complex structure of the dataset and fuse node features using a graph autoencoder (GAE), effectively extracting the latent features.

*Eigenvalue transfer:*

4 $$ X^{\prime } = X*A*A $$

*Network structure:*

5 $$ Z = f(X,A) = Leaky\,ReLU((Leaky\,ReLU(XAW^{(0)} - b^{(0)} )AW^{(1)} - b^{(1)} ) $$

*Loss function:*

6 $$ J(W,b) = \sum {(L} (X^{{\prime }} ,Z)) = \sum {\left\| {X^{{\prime }} - Z} \right\|}^{2} $$

**Figure 3:**
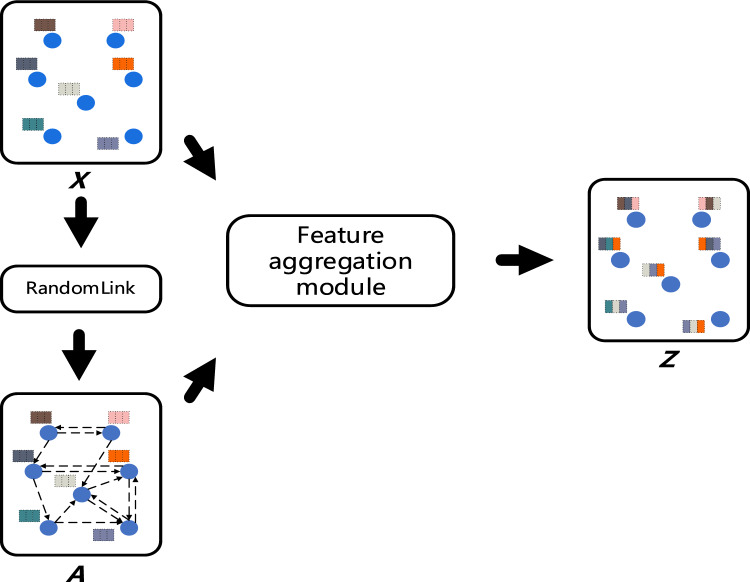
Feature fusion.

During the training process of the Graph Autoencoder (GAE), we utilize the gradient descent algorithm to update the GAE weights W(0), W(1), and bias vectors b(0), b(1). The structure of feature fusion model is shown in Fig. 3.

In this study, we employ a variety of base detectors, including traditional methods, deep learning-based methods, and the latest graph neural network-based methods. Our objective is to provide a more comprehensive and accurate solution for outlier detection.

The selection of base detectors is a crucial and challenging aspect in ensemble learning, as their performance directly impacts the performance of the integrated model. Ideally, the base detectors should demonstrate high individual performance and complement each other. They should show different performance in various subspaces of data features. However, accurately predicting the applicability range of a base detector in practical scenarios is difficult due to the unknown, variable, and high-dimensional nature of data feature distributions. Therefore, it is necessary to consider their diversity and complementarity when selecting base detectors to improve the generalization and stability of the integrated model.

### Bearing fault diagnosis through ensemble learning

To enhance the convergence speed of the algorithm, it is crucial to normalize the output of the base detectors. Normalization is a vital pre-processing step as it addresses the issue of varying magnitudes among different base detectors, which makes direct comparison and combination challenging. Equation (7) illustrates the normalization equation. This procedure entails subtracting the data by its mean μ and dividing it by the variance σ, thereby converting the processed data into a standard normal distribution.

7 $$ x = (x - \mu )/\sigma $$

**Figure 4:**
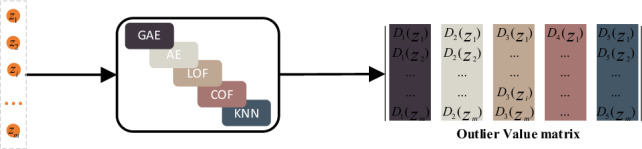
Ensemble learning.

The ***Z***-matrix, obtained by aggregating the features of neighboring nodes through GAE, serves as input for each base detector mentioned above. This process facilitates the construction of an integrated learning model with diversity. The output of each base detector in the integrated learning model is then normalized to generate an outlier matrix. The structure of the ensemble learning model is depicted in Fig. 4.

where *z*_*i*_ denotes the *i*-th object in the matrix ***Z*** generated from the original dataset *X* after GAE feature fusion. ***D*** denotes the set of base detectors, ***D***_***c***_ denotes the cth base detector in the set of base detectors. ***D***_***c***_***(z***_***i***_***)*** denotes the outlier of ***z***_***i***_ at the cth base detector. iter denotes the column position of each base detector in ***D***, init() means to initialize the base detector, train() denotes the training base detector by ***Z***_***m*×*n***_.

**Algorithm 2:**
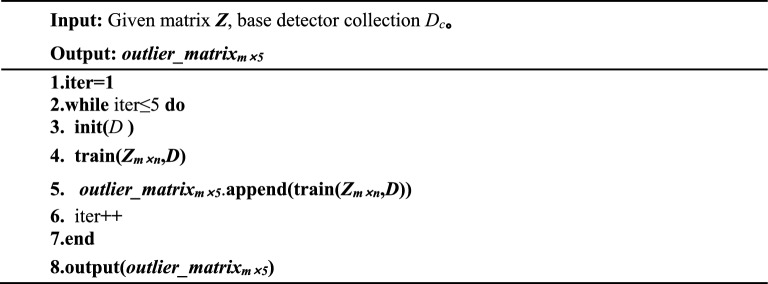
Outlier value matrix generation.

#### Marking outlier levels

Currently, the predominant research trend in outlier detection focuses on unsupervised learning. However, the lack of labeled data presents a challenge in accurately assessing the disparity between the predicted outcome of the detector and the extent of unlabeled outliers. To address this issue, we propose a hybrid data-based approach that combines unlabeled normal and outlier data for training. In this method, points with higher degrees of outliers are more likely to be identified as outliers after the model learns the features and generates outputs. Algorithm 2 utilizes basic detectors to detect each object in the ***Z*** matrix and produces diverse outliers as output. We then select the maximum value from these outliers as a measure of the degree of outlier present in the data. Specifically, the labeled outliers are calculated as follows:

8 $$ Label(z_{i} ) = \max \{ D_{1} (z_{i} ),D_{2} (z_{i} ), \ldots ,D_{5} (z_{i} )\} $$

**Figure 5:**
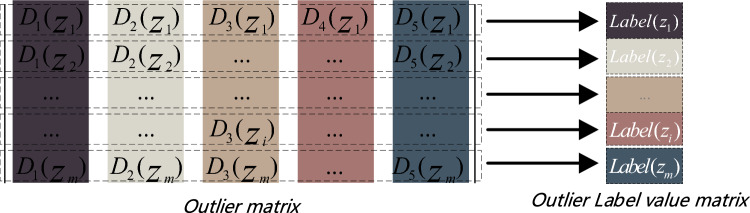
Mark outlier degree.

The matrix of label outliers for all data in *Z*_*m*×*n*_ is calculated from Eq. (8) and is shown in the Fig. 5.

#### Local area construction

The BFDGE algorithm requires the construction of local regions due to the correlation between data objects in a dataset. It uses the detection capability of a base detector on neighboring objects to estimate its detection capability on a specific object. Hence, the algorithm calculates the detection capability of the base detector over a local region. To achieve this, the BFDGE algorithm divides the labelled outliers into clusters and identifies the cluster to which the target object belongs. We use the KNN algorithm to calculate the Euclidean distance between node ***z***_***i***_ and its surrounding neighboring nodes, and then determine the k nearest neighbor nodes of ***z***_***i***_ according to the magnitude of the Euclidean distance. These nearest neighbor nodes form the set of neighbors of zi, as shown in Eq. (9):

9 $$ \Omega_{i = } \{ p_{i} |\mathop {\arg \min }\limits_{i = 1,2, \ldots ,k} ||z_{i} - p_{i} ||\} $$

We use the KNN algorithm to find the neighboring ***k*** data and deposit them into the neighborhood cluster ***Ω***. It is important to note that the choice of ***k*** values affects the creation of the neighborhood clusters. First, as the ***k***-value increases, the number of nearest neighbors to be computed increases, thus increasing the computational complexity of the algorithm. Secondly, the size of ***k***-value directly affects the accuracy of prediction. When the ***k*** value is small, the algorithm will be more sensitive and may over-fit the data, while when the ***k*** value is large, the algorithm will be smoother and may ignore the detailed features of the data.

#### Combination of base detectors

After determining the neighborhood cluster ***Ω*** of object ***z***_***i***_ in ***Z***, we calculate the detection capability of all base detectors on this local area with the aim of selecting the combination of base detectors with strong detection capability for ***z***_***i***_**.**

We can obtain the outliers corresponding to each data ***p***_***i***_ in the neighborhood cluster ***Ω*** from the already obtained ***outlier_matrix***. The outliers corresponding to each data *p*_*i*_ are stored in the matrix. As shown in Eq. (10):

10 $$ O_{{k \times 5}}  = \left| {\begin{array}{*{20}l}    {D_{1} (p_{1} )} \hfill & {D_{2} (p_{1} )} \hfill & {D_{3} (p_{1} )} \hfill &  \cdots  \hfill & {D_{5} (p_{1} )} \hfill  \\    {D_{1} (p_{2} )} \hfill &  \cdots  \hfill &  \cdots  \hfill &  \cdots  \hfill & {D_{5} (p_{2} )} \hfill  \\     \cdots  \hfill &  \cdots  \hfill & {D_{3} (p_{i} )} \hfill &  \cdots  \hfill &  \cdots  \hfill  \\     \cdots  \hfill &  \cdots  \hfill &  \cdots  \hfill &  \cdots  \hfill &  \cdots  \hfill  \\    {D_{1} (p_{k} )} \hfill &  \cdots  \hfill &  \cdots  \hfill &  \cdots  \hfill & {D_{5} (p_{k} )} \hfill  \\   \end{array} } \right| $$

The label outliers corresponding to each object ***p***_***i***_ in the neighborhood cluster ***Ω*** can be obtained from the label outlier matrix ***Label***_***m*×*1***_ obtained from Eq. (9), and the label outliers corresponding to each*** p***_***i***_ are stored in the matrix. ***Q***_***k*×*1***_ As shown in Eq. (11):

11 $$ Q_{{k \times 1}}  = \left|\begin{array}{*{20}c}    {Label(p_{1} )}  \\     \cdots   \\    {Label(p_{i} )}  \\    \cdots   \\    {Label(p_{k} )}  \\   \end{array} \right| $$

After that, we use the cosine similarity to calculate the difference between ***O***_***k*×*5***_ and ***Q***_***k*×*1***_ to derive the detection capability of the base detector ***ϑ***_***r,i***_. The better the detection capability of the base detector on*** p***_***i***_, the higher the cosine similarity between its output value and the ***p***_***i***_ label outliers. The calculation is shown in Eq. (12):

12 $$ \vartheta_{r,i} = \frac{{D_{r} (p_{i} ) \times Label(p_{i} )}}{{||D_{r} (p_{i} )|| \times ||Label(p_{i} )||}} $$

With the above formula, we can get these ***ϑ***_***r,i***_. for each object ***p***_***i***_ in ***Ω***; then sort these ***ϑ***_***r,i***_ in descending order and obtains base detectors with strong detection capability for *p*_*i*_ by selecting the top-S neighborhood clusters.

Similarly, for all objects in ***Ω***, we select the base detector with strong detection power on the local region ***Ω*** to detect the neighborhood cluster ***z***_***i***_ and output (***num_local* × *s***) outliers. Finally, we calculate the average of these (***num_local* × *s***) outliers as the final outliers of the neighborhood cluster ***z***_***i***_.

In this process, we ranked the ***ϑ***_***r,i***_**.** in descending order and the top *n* of ***num_detector***’s had high similarity scores.

13 $$ s = [num\_detector \times \theta ] $$

In the Eq. (13), the ***θ*** represents the outlier ratio. For the set ***S***, we sort the elements according to their size, where a higher value of *S* means that the element is more likely to be an outlier. Then, we select the top *n* objects in *S* as the final outliers.

## Experiments

In this section, we provide a detailed description of the experimental design and results. Our objective was to validate the effectiveness of the method in detecting bearing faults. To achieve this, we conducted a comparative experiment, comparing our method with several state-of-the-art algorithms. The source code of the model was implemented using MATLAB R2021A. The experimental hardware setup consisted of a Ryzen 7 5800H 3.20 GHz CPU and 16 GB RAM, while the operating system environment was Microsoft Windows 11 Professional.

### Introduction of the dataset

The test setup for Dataset 1 included a 2 hp motor, torque transducer/encoder, dynamometer, and control electronics. The motor shaft was supported by the test bearings. To induce failure, the motor bearings were manufactured using electric discharge machining (EDM). Fractures of 0.1778 mm, 0.3556 mm, and 0.5334 mm in diameter were intentionally introduced in the inner race, rolling element (ball), and outer race, respectively. The faulty bearing was then reinstalled into the test motor, and vibration data was recorded at 0 motor load (motor speed of 1797 RPM). The bearing used for this test was SKF 6250, positioned at the drive end. Digital data was collected at a rate of 12,000 samples per second, and for the drive end bearing failure, data was collected at a rate of 48,000 samples per second. Speed and horsepower data were collected using a torque sensor/encoder and recorded manually. Table 1 provides a summary of the CWRU datasets.

**Table 1:** Summary of CWRU datasets.

	Fault type		Sample number	
Inner race fault	Normal		800	
	Inner race fault	0.1778 mm	0.3556 mm	0.5334 mm
		20	20	20
Ball fault	Normal		800	
	Ball fault	0.1778 mm	0.3556 mm	0.5334 mm
		20	20	20
Out race fault	Normal		800	
	Outer race fault	0.1778 mm	0.3556 mm	0.5334 mm
		20	20	20

Dataset 2 was obtained from the bearing dataset at Xi’an Jiaotong University. The experiments used LDK UER204 bearings, and the degradation vibration signals were measured under various operating conditions. The sampling frequency during data acquisition was set to 25.6 kHz, with a sampling interval of 1 min and a duration of 1.28 s for each sampling. To assess the algorithm’s robustness, a set of bearing degradation data was selected for each of the three different operating conditions. Table 2 provides the distribution of the XJTU datasets.

**Table 2:** Summary of XJTU datasets.

	Work condition (RPM, radial force/kN)	Fault type	Sample number
(1-1)	(2100/12)	Normal	800
		Inner race fault	60
(2-1)	(2250/11)	Normal	800
		Ball fault	60
(3-1)	(2400/10)	Normal	800
		Outer race fault	60

We calculated 23 indicators in the time and frequency domains for the samples in the datasets. These indicators are more convenient for downstream tasks and help improve the quality and accuracy of the data. Additionally, they reduce modeling errors and biases, and enhance the accuracy and interpretability of the model when compared to the original dataset.

The sequence x(n) represents a set of discrete data points, while its arithmetic mean is represented by. The size of the sequence, or the number of data points, is denoted as *N*. The sequence *x*_*i*_(*n*), where i ranges from 0 to 2^*j*^-1, denotes the decomposition coefficient sequence of the ith frequency band using WPD, a decomposition method that operates at level j,Wavelet Packet Decomposition (WPD) is an extension of the wavelet transform that offers a more comprehensive signal analysis. It achieves this by decomposing the signal into more detailed frequency bands compared to traditional wavelet analysis. WPD is highly regarded in vibration signal analysis due to its effectiveness in extracting characteristic fault frequencies from noisy signals. This leads to an improved accuracy in fault diagnosis for rotating machinery.In the context of bearing fault diagnosis, WPD enables the extraction of subtle features from bearing vibration signals. These features indicate the early stages of faults, which may not be detectable using other methods. The ability of WPD to perform time–frequency analysis makes it particularly suitable for diagnosing mechanical faults in bearings under varying load and speed conditions. This is because the frequency content of the signal changes over time in such cases. Meanwhile, IMFi(n) refers to the ith data sequence resulting from EEMD, a separate decomposition method that operates at level NI. EEMD, short for Ensemble Empirical Mode Decomposition, is an advanced signal processing technique that improves upon the Empirical Mode Decomposition (EMD) method. EEMD addresses the issue of mode mixing observed in EMD by introducing white noise to the data in multiple iterations. This iterative process enhances the robustness and reliability of the decomposition, enabling more accurate analysis of complex, non-linear, and non-stationary signals. Due to its adaptability and efficiency in handling real-world complex data, EEMD finds extensive applications in various fields including signal processing, time-series analysis, and even environmental and medical data analysis.

Based on Table 3, the index is calculated for each sample. Four steps are required.

Nine-time domain indexes are calculated as follows:
  14 $$ I = \left[ {I_{1} ,I_{2} ,I_{3} ,I_{4} ,I_{5} ,I_{6} ,I_{7} ,I_{8} ,I_{9} } \right] $$
*E*_WPD_ is obtained by calculating WPD energy (parameters *j* = 3 and wavelet Db20).
  15 $$ W_{WPD} = \left[ {E_{WPD}^{1} ,E_{WPD}^{2} ,E_{WPD}^{3} ,E_{WPD}^{4} ,E_{WPD}^{5} ,E_{WPD}^{6} ,E_{WPD}^{7} ,E_{WPD}^{8} } \right] $$
(3): EEMD energy is calculated to obtain a dataset as follows:
  16 $$ W_{EEMD} = \left[ {E_{EEMD}^{1} ,E_{EEMD}^{2} ,E_{EEMD}^{3} ,E_{EEMD}^{4} ,E_{EEMD}^{5} ,E_{EEMD}^{6} } \right] $$
(4): *I*, *W*_*WPD*_, *W*_*EEMD*_ are combined into a dataset as follows:
  17 $$ X = \left[ {I,W_{WPD} ,W_{EEMD} } \right] $$

**Table 3:** Indexes and the calculation formulas.

Indexes	Formulas
1. Standard deviation	$I_{1} = \sqrt {\sum\nolimits_{n = 1}^{N} {(x(n) - \overline{x} )}^{2} /N}$
2. Peak	$I_{2} = \max |x(n)|$
3. Skewness	$I_{3} = \sum\nolimits_{n = 1}^{N} {(x(n) - \overline{x} )^{3} } /(N - 1)I_{1}^{3}$
4. Kurtosis	$I_{4} = \sum\nolimits_{n = 1}^{N} {(x(n) - \overline{x} )^{4} } /(N - 1)I_{1}^{4}$
5. Root mean square	$I_{5} = \sqrt {\sum\nolimits_{n = 1}^{N} {x(n)}^{2} /N}$
6. Crest factor	$I_{6} = I_{2} /\sqrt {\sum\nolimits_{n = 1}^{N} {x(n)}^{2} /N}$
7. Square	$I_{7} = I_{2} /(\sum\nolimits_{n = 1}^{N} {\sqrt {x(n)} } /N)^{2}$
8. Shape factor	$I_{8} = \sqrt {N\sum\nolimits_{n = 1}^{N} {x(n)^{2} } } /\sum\nolimits_{n = 1}^{N} {|x(n)|}$
9. Impulse factor	$I_{9} = \max |x(n)|/(\sum\nolimits_{n = 1}^{N} {{|}x(n)} {|}/N)$
10. WPD energy	$I_{10} = \sum\nolimits_{i = 1}^{N} {|x_{i} (n)|}^{2} /\sum\nolimits_{i = 0}^{{2^{j - 1} }} {\sum\nolimits_{n = 1}^{N} {|x_{i} (n)|} }^{2}$
11. EEMD energy	$I_{11} = \sum\nolimits_{n = 1}^{N} {|IMF_{i} (n)|}^{2} /\sum\nolimits_{i = 1}^{NI} {\sum\nolimits_{n = 1}^{N} {|IMF_{i} (n)|} }^{2}$

We calculated 23 indicators in the time and frequency domains for the samples in the datasets. These indicators are more convenient for downstream tasks and help improve the quality and accuracy of the data. Additionally, they help reduce modeling errors and biases, and enhance the accuracy and interpretability of the model compared to the original dataset.

**Figure 6:**
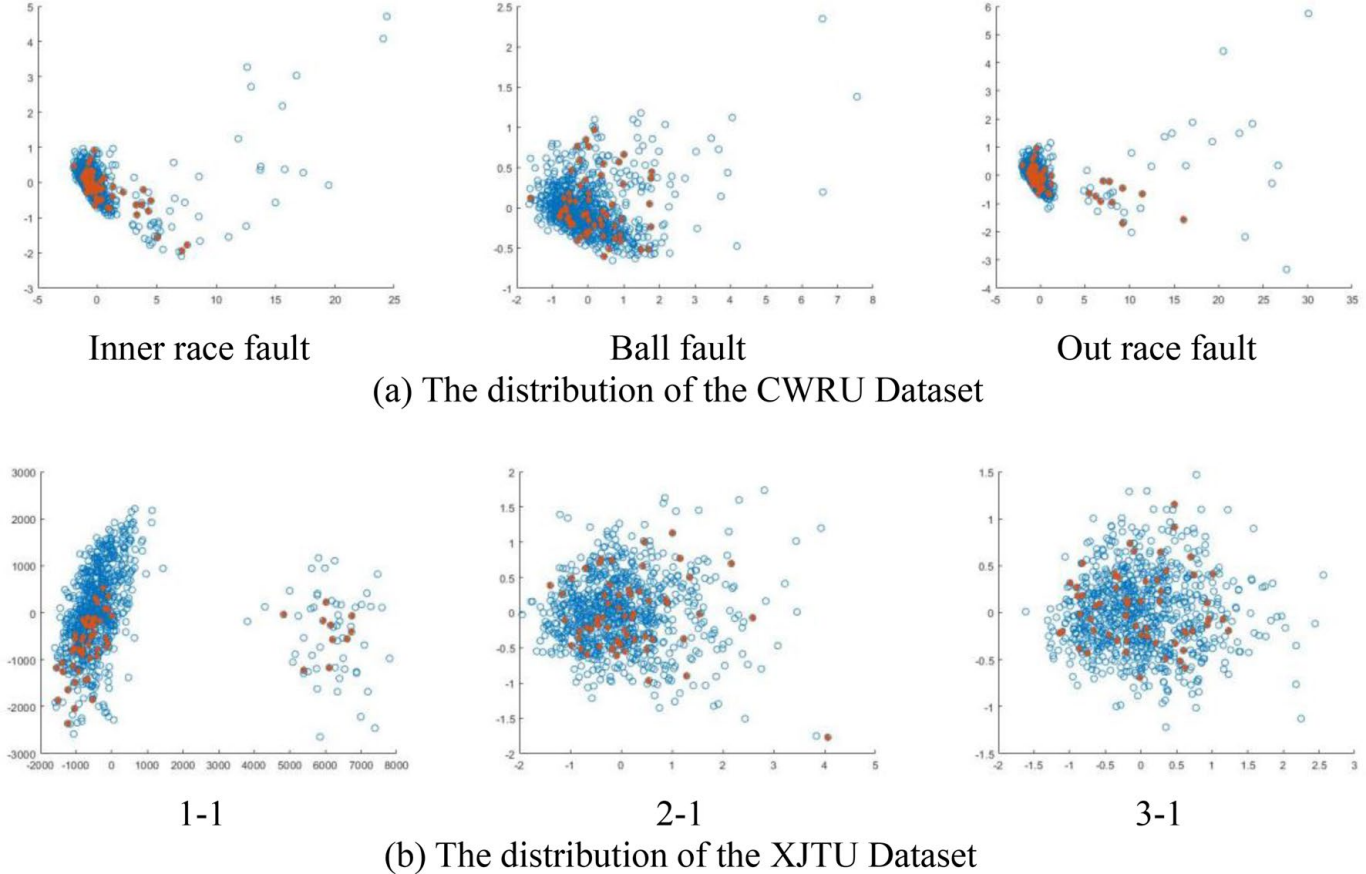
The distribution of the selected dataset.

The distribution of the selected dataset is illustrated in the accompanying Fig. 6,we performed PCA dimensionality reduction on the original dataset, where normal objects are represented by blue hollow circles and abnormal objects are represented by red solid circles. It is evident that a significant portion of the outliers are intermingled with the normal data, making their differentiation challenging.

### Comparison methods

In this paper, we investigate the problem of bearing fault detection and approach it as an anomaly detection problem in the field of artificial intelligence. To validate the effectiveness of our proposed algorithm, we conducted comparison experiments using state-of-the-art outlier detection algorithms. To ensure robust conclusions, we selected and compared five different types of state-of-the-art outlier detection algorithms. These algorithms are commonly used in the field of outlier detection and have been extensively studied in the literature, demonstrating their effectiveness. It's noteworthy to mention that the GAN-based approach proposed by Du et al.^49^ exhibits greater novelty. By comparing our algorithm with these established methods, we aim to evaluate its performance, strengths, and weaknesses, and further enhance and optimize it. The experimental results demonstrate that our algorithm performs exceptionally well across all metrics, confirming its effectiveness and feasibility in detecting bearing faults. Table 4 presents a list of the comparison algorithms and their respective types.

**Table 4:** Comparison algorithm.

Type of algorithm	Acronym of algorithm
Neural network-based	AE
Local outlier factor-based	LOF
Connective-based	COF
Distance-based	KNN
Graph neural network-based	GAE
Generative adversarial network-based	GUOD

Since the experiments involve multiple algorithms that require different hyperparameters to be set, Table 5 is used to describe in detail the parameter settings used for each algorithm in the experiments.

**Table 5:** Parameter setting.

Algorithms	*K* (Number of nearest neighbors)	Learning rate	Number of iterations	Number of layers
BFDGE	2–100	0.0001–0.002	10–100	3
AE	–	0.0001–0.002	10–100	3
LOF	2–100	–	–	–
COF	2–100	–	–	–
KNN	2–100	–	–	–
MO-GAAL	–	0.0001–0.002	10–100	3
GUOD	–	0.0001–0.002	10–100	3

AUC (Area Under the Curve) is a widely used metric for evaluating the performance of binary classification models. It measures the average ability of the classifier to distinguish between positive and negative cases by calculating the area under the Receiver Operating Characteristic (ROC) curve. AUC is considered one of the most important metrics for assessing the prediction accuracy of a model.

False Alarm Rate (FAR), also known as the false positive rate, measures the probability of the model misclassifying negative cases as positive cases. It is an important metric for evaluating the extent to which the classifier incorrectly classifies positive cases in samples of negative cases.

Detection Rate (DR), also known as the true positive rate, measures the probability of the model correctly classifying a positive case as a positive case. It is an important indicator of the classifier's ability to correctly classify positive cases in a sample.

Accuracy (ACC) is the ratio of samples correctly classified by the classifier to the total number of samples. It is a crucial metric for evaluating the overall performance of the classifier.

A Confusion Matrix is a table that presents the prediction results of a binary classification model. It consists of four values: True Positive (TP), False Positive (FP), True Negative (TN), and False Negative (FN). TP represents the number of samples correctly predicted as positive cases, FP represents the number of samples incorrectly predicted as negative cases, TN represents the number of samples correctly predicted as negative cases, and FN represents the number of samples incorrectly predicted as positive cases. By utilizing the confusion matrix, we can calculate important metrics such as DR, FAR, ACC, and AUC. The formula for calculating AUC is shown in Eq. (19).

18 $$ AUC = \frac{{\sum\nolimits_{i = 1}^{n - 1} {(x_{i + 1} - x_{i} ) \times (y_{i + 1} - y_{i} )} }}{2} $$

*x*_*i*_ and *y*_*i*_ are the horizontal and vertical coordinates of the i-th sample, respectively, and *i* is a positive integer; *n*_+_ and *n*_*−*_ are the number of positive and negative cases, respectively; and n is the total number of samples. ACC and DR and FAR are shown in (19), (20), and (21):

19 $$ ACC = \frac{TP + TN}{{TP + TN + FP + FN}} $$

20 $$ DR = \frac{TP}{{TP + FN}} $$

21 $$ FAR = \frac{FP}{{TN + FP}} $$

### Experimental results

In this section, we present a visual comparison of the AUC performance of our algorithm with six other algorithms for bearing fault diagnosis. The comparison is shown on a bar chart. Additionally, we provide a table that further highlights the superior performance of our algorithm on the remaining four metrics.

Table 6 display the results of the BFDGE algorithm applied to six real bearing fault diagnosis datasets, in comparison to six other algorithms, based on four performance metrics. The table illustrates that BFDGE achieves the highest AUC and ACC values across all six datasets. Moreover, BFDGE demonstrates a more substantial enhancement in DR and FAR on the six datasets, respectively, compared to the second-place algorithm. Additionally, BFDGE attains the lowest FAR values on all three datasets. BFDGE excels in detecting faulty samples that are mixed with normal samples, which poses a challenge for traditional distance, density, and model-based algorithms. Our approach utilizes graph neural networks to aggregate neighboring node features and employs integrated learning to enhance the robustness of the detection results, thereby yielding superior and more consistent performance.

**Table 6:** Experimental results on real-world datasets.

Dataset	AUC						
	BFDGE	AE	LOF	COF	KNN	MO-GAAL	GUOD
(a) AUC score for each algorithm on real world datasets							
Inner race fault	**0.97**	0.93	0.71	0.92	0.69	**0.95**	0.93
Ball fault	**0.98**	0.87	0.73	0.83	0.71	0.82	**0.96**
Out race fault	**0.98**	0.91	0.94	0.60	0.87	0.93	**0.97**
1-1	**0.98**	0.94	0.47	0.53	0.97	0.59	0.73
2-1	**0.74**	0.55	0.52	0.49	0.52	0.48	0.66
3-1	**0.95**	0.46	0.58	0.56	0.57	0.63	0.61
(b) Accuracy score of each algorithm on real world datasets							
Inner race fault	**0.97**	0.93	0.71	0.92	0.69	0.95	**0.97**
Ball fault	**0.98**	0.87	0.73	0.83	0.71	0.82	**0.98**
Out race fault	**0.98**	0.91	0.94	0.60	0.87	0.93	0.97
1-1	**0.98**	0.94	0.87	0.86	0.97	0.95	0.94
2-1	0.74	0.55	0.52	0.49	0.52	0.48	**0.90**
3-1	**0.95**	0.86	0.87	0.85	0.88	0.93	0.90
(c) Detection rate of each algorithm on real world datasets (%)							
Inner race fault	**98.33**	80.00	55.00	83.33	30.00	86.67	83.33
Ball fault	**96.67**	48.33	40.00	48.33	36.67	46.67	93.33
Out race fault	**98.33**	66.67	73.33	40.00	63.33	70.00	86.67
1-1	**96.67**	70.00	10.00	6.67	81.67	46.67	61.67
2-1	**66.67**	5.00	3.33	10.00	16.70	7.50	33.33
3-1	**90.00**	5.00	10.00	8.33	6.67	0.93	28.33
(d) False alarm rate for each algorithm on real world datasets (%)							
Inner race fault	**0.75**	1.50	3.25	1.25	3.75	1.00	1.25
Ball fault	**0.50**	3.87	4.50	3.87	4.75	4.00	**0.50**
Out race fault	**0.25**	2.50	0.94	4.50	2.75	2.25	1.00
1-1	**1.50**	2.25	6.75	7.00	1.38	3.25	2.88
2-1	**4.25**	7.13	7.25	7.50	7.50	10.00	5.00
3-1	**2.25**	7.13	6.75	6.88	7.00	4.25	5.38

Significant values are in bold.

### Robustness experiments

**Figure 7:**
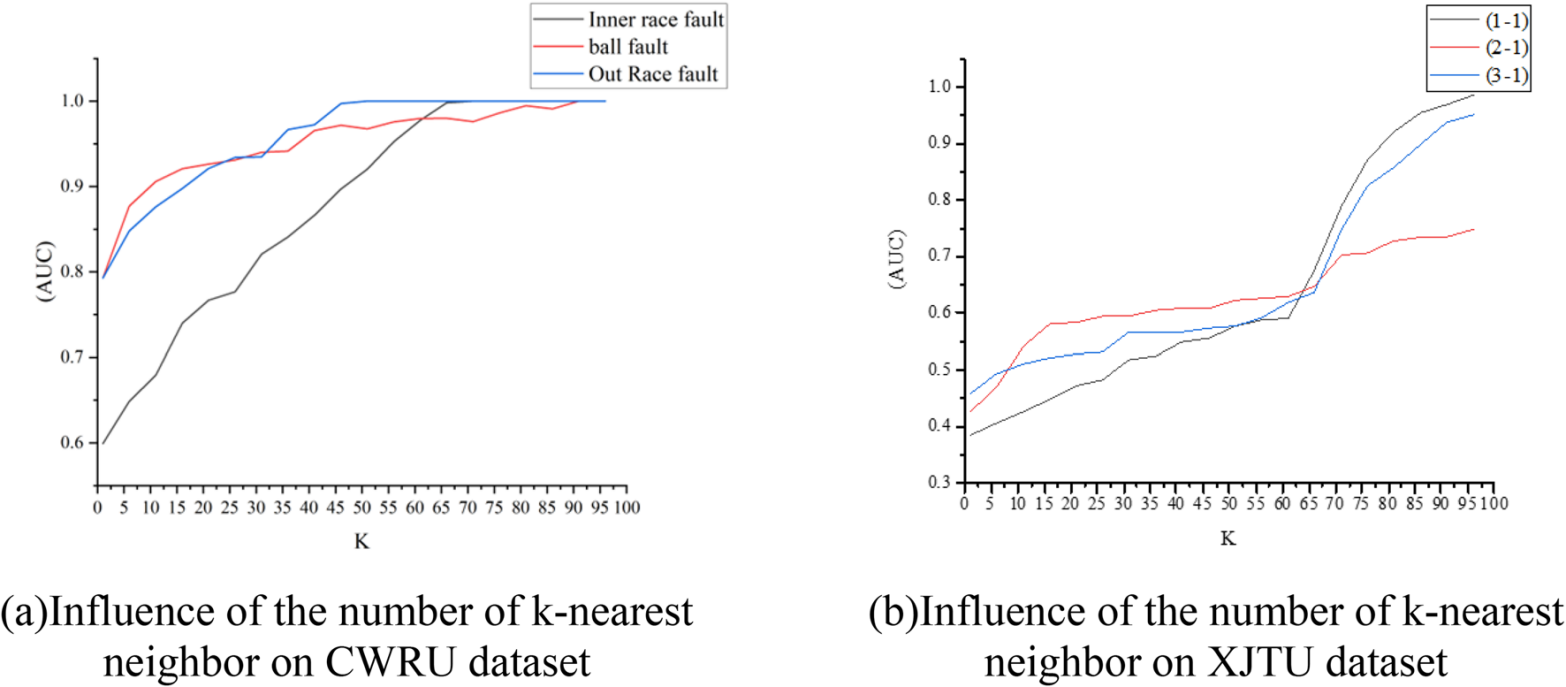
Influence of the number of k-nearest neighbor on BFDGE.

BFDGE involves various parameters that affect its performance. These parameters include the number of base detectors, the number of node neighbors in RandomLink, the number of nodes in the local region of each node, the number of hidden layer neurons in the graph neural network, and the learning rate. For our in-depth study, we focused on two parameters that have a significant impact on the detection results: the number of node neighbors (***k***) and the number of hidden layers in RandomLink. We conducted 20 sets of experiments on three datasets to investigate the effects of these parameters on the performance of BFDGE. The experimental results are as follows:

As depicted in Fig. 7, the Area Under the Curve (AUC) of the BFDGE exhibits a gradual increase with the increment in the number of nodes (***k***) connected to each node in the RandomLink, until it reaches a stable state. This observation suggests that the limited aggregation of random node features per node, due to a restricted number of randomly aggregated nodes, hinders the BFDGE's ability to effectively differentiate between normal and faulty objects. However, with the gradual increase in ***k***, the AUC values of BFDGE also progressively increase on the three datasets, ultimately stabilizing at the highest attained AUC.

**Figure 8:**
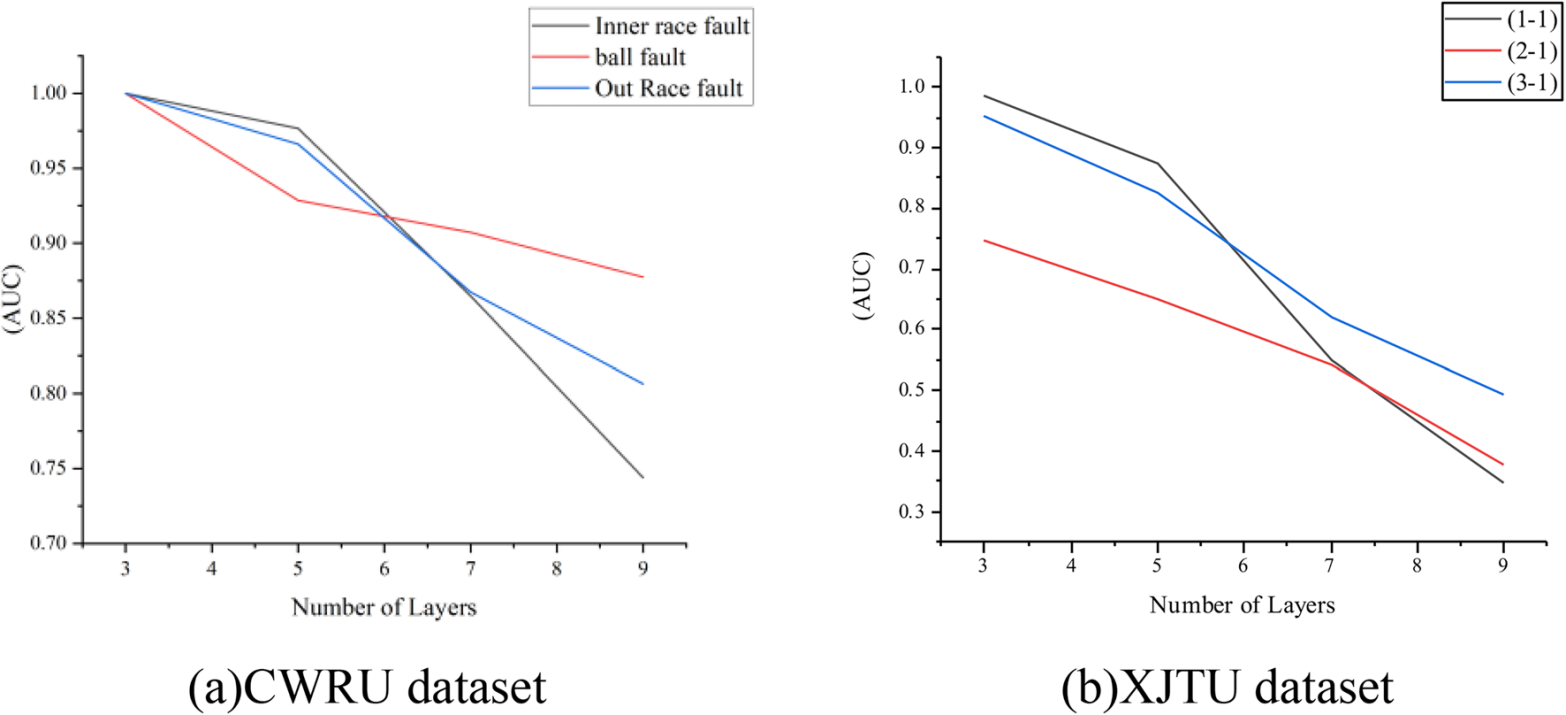
Influence of the number of layers on BFDGE.

Based on the observation of Fig. 8, it is evident that increasing the number of layers in the graph neural network to 3 results in the highest AUC value. As the network's depth increases, the feature values between objects become increasingly similar, leading to similar reconstruction errors in the output layer. This similarity makes it challenging to differentiate between normal and abnormal objects, resulting in over-smoothing.

## Conclusion

This paper presents a novel fault detection method for bearing faults using a graph neural network based on ensemble learning. Early faults in bearings often have small amplitude and low intensity characteristic signals, making them inconspicuous, random, and easily masked by system interference and noise. To address this issue, we propose a combinatorial method that converts the original dataset into a graph dataset and utilizes a feature aggregation module to aggregate neighboring node features. Subsequently, unsupervised bearing fault detection is performed using integrated learning. The method involves converting vibration signals into graphs to establish correlations between initially independent signals. The dataset, along with the corresponding graphs, is then inputted into the feature aggregation module for training, enabling fault detection through a new integrated learning strategy. Through detailed comparisons with existing algorithms, we demonstrate that the proposed method successfully detects faulty objects within normal object regions or around dense clusters. In future work, we intend to explore new compositional methods, graph neural networks, and loss functions to achieve even more satisfactory and stable results.

## Data Availability

The datasets generated and/or analyzed during the current study are available in the CWRU repository, https://engineering.case.edu/bearingdatacenter/welcome.
